# Phenoconversion and rural pain inequities: a precision-informed roadmap to advance equity for rural older adults

**DOI:** 10.3389/fpain.2026.1883083

**Published:** 2026-10-14

**Authors:** Alexis K. Hall, Emily J. Cicali, Denise Schentrup, Andrew H. Dubin

**Affiliations:** 1 College of Nursing, University of Florida, Gainesville, FL, United States; 2 Department of Pharmacotherapy and Translational Research, College of Pharmacy, University of Florida, Gainesville, FL, United States; 3 Department of Physical Medicine and Rehabilitation, College of Medicine, University of Florida, Gainesville, FL, United States

**Keywords:** CYP2D6, drug interactions [MeSH], geriatric (aging), pain – drug therapy, phenoconversion, polypharmacy (MeSH), precision medicine, rural

## Abstract

Inequities in geriatric pain management persist despite three decades of research, disproportionately affecting older adults in rural communities. Structural barriers, multimorbidity, polypharmacy, and phenoconversion that alters functional drug metabolism, contribute to variable analgesic response and medication-related harm. The underrecognized mechanism, phenoconversion, is particularly consequential for patients prescribed CYP2D6-dependent analgesics, where drug-drug-gene interactions can render enzyme-dependent opioids ineffective or precipitate toxicity. Case-based illustrations demonstrate how unrecognized phenoconversion can lead to avoidable suffering and safety risks. Additionally, an implementation vignette highlights opportunities for integrating pharmacogenomics, shared decision making, and interoperable electronic care plans into rural primary care. These tools help ensure that critical information, such as patient goals, caregiver perspectives, and functional priorities, accompany medication decisions across transitions of care where phenoconversion risks may often be raised. Building on these insights, we propose a precision-informed roadmap calling for clinical decision infrastructure, telehealth-enabled specialty access, workforce development, and system-level support. When combined with person-centered care approaches, precision health care offers a promising pathway toward safer, more effective, and more equitable pain management for rural older adults.

## 1 Introduction

Inequities in geriatric pain management remain entrenched. An estimated 40% of those over 65 years-old experience unrelieved pain (1), placing them at risk for delayed recovery and medication-related harm. Older adults are disproportionately represented in rural communities compared to urban areas (2). In a recent web-based survey of rural stakeholders, 17.7% identified chronic pain among the top 20 most important priorities for Healthy People 2030 (3). Severe pain (4), along with chronic pain, is more prevalent among rural populations (1, 4). These disparities reflect the combined influence of structural barriers and biological complexity, including often unrecognized effects of multimorbidity, polypharmacy, potentially inappropriate medications (PIMS), and metabolic variability (1, 4–7). Rural Health Clinics (RHCs), which serve as essential access points for medically underserved populations, operate under reimbursement and workflow constraints that limit the adoption of precision-informed pain management strategies to those that can be embedded within existing visit types (8). Cash-based alternatives remain inaccessible for many patients in RHCs. Policy alignment with emerging precision health capabilities continues to lag, slowing adoption and widening the gap between scientific advances and real-world implementation in rural primary care (9).

Within this landscape, phenoconversion represents an underappreciated mechanism (10–14). Drug-drug-gene interactions that alter the functional metabolic activity of cytochrome P-450, CYP2D6, potentially render commonly prescribed opioids ineffective or unsafe, and may contribute to avoidable suffering and non-guideline-concordant care (10–14). Case-based observations illustrate how unrecognized phenoconversion shapes analgesic response as well as safety in rural older adults, underscoring the potential need for greater clinical awareness and system-level support. An implementation-focused vignette points to the creation of a policy-aligned roadmap to embed precision health into rural primary care, as is called for by the literature (14, 15).

It is important to note that while phenoconversion is clinically well-established (5, 6, 10–14), its contribution to rural pain inequities remains a plausible but incompletely characterized pathway. The roadmap presented here, therefore, reflects an implementation-focused proposal rather than an empirically validated solution. Three elements: 1) evidence-supported pharmacological mechanisms underlying phenoconversion; 2) plausible interactions between these mechanisms and rural structural disadvantages; and 3) proposed system-level interventions, form the basis for the proposal to strengthen equity in geriatric pain management by addressing both biological contributors to treatment variability and contextual drivers of rural health disparities.

## 2 Historical evolution of pain disparities research

Pain disparities research has developed through the experience of three generations of research and is arriving at a fourth generation that operationalizes and evaluates transformative, equity-driven solutions (16). Grounded in documentation of inequities (Generation 1), informed by underlying biopsychosocial mechanisms (Generation 2) and early evaluation of the effectiveness and accessibility of multidimensional, integrative, life course approaches (17) that address diverse social identities and contexts (Generation 3), the emerging, justice-oriented fourth generation aims to inclusively transform, “disparities into equities” (16). Applied to the rural setting, fourth generation fundamental change could empower older adults in their care by addressing overlooked root causes of persistent pain (10–13). This includes structural context and systemic forces that shape pain outcomes (14–16, 20), such as the disproportionate reliance on primary care clinicians in rural settings, where infrastructure, staffing, and reimbursement support required for guideline-concordant pain management are often lacking (17–19).

### 2.1 Structural and systemic drivers of disparities

Structural inequities remain central drivers of pain disparities among rural older adults, influencing those who receive timely diagnosis, access to multimodal therapies, and the level of clinical support needed to manage complex conditions (4, 20). Rural communities face persistent shortages of pain specialists, geriatricians, behavioral health clinicians, and pharmacists with pharmacogenomics (PGx) expertise, though rural residents are more often exposed to medications with PGx guidance (4, 21). Geographic isolation and transportation barriers delay escalation of care, while digital inequities limit the reach of telehealth-enabled pain interventions (4). Rurality is correlated with higher rates of opioid prescriptions (4, 20), and those with depression and/or comorbidities have been found to be more likely to receive opiates (4). Furthermore, there is a documented rural-urban disparity in age-adjusted mortality (22). These findings likely reflect the fragmented care models that struggle to address multimorbidity, polypharmacy, and dynamic, age-related physiology.

### 2.2 Intra-group variability in geriatric pain

Although older adults are often treated as a homogenous group, they exhibit substantial intra-group variability in pain experience, treatment response, functional priorities, and social context (1, 4–6, 16). This heterogeneity reflects differences in biology, comorbidity burden, and lived experience, which shape analgesic selection, effectiveness and risk (4–6, 23). Genetic differences in drug-metabolizing enzymes, epigenetic changes shaped by life-course exposures (24–26), frailty and age-related changes in pharmacokinetics and pharmacodynamics, as well as polypharmacy, alter medication effects in clinically meaningful ways (5, 6, 13, 27). Psychosocial and behavioral factors, including coping strategies, social support, and cultural expectations, also influence pain reporting and treatment preferences (1, 4, 16). Precision medicine is currently underutilized but provides a means to address this heterogeneity by tailoring pain management to an individual's biological, environmental, and contextual factors (19, 28).

## 3 Phenoconversion, an underrecognized contributor to pain disparities

Precision medicine, encompassing PGx-guided precision prescribing and an understanding of phenoconversion, can improve pain outcomes by aligning medication selection and dosing with an individual's functional metabolic profile, referred to as clinical phenotype (5, 6, 10–14, 28–31). Clinical phenotype can diverge from the individual's genotype-predicted phenotype, due to drug-drug-gene interactions, inflammation, or organ dysfunction. Precision medicine is expected to be especially relevant in geriatric pain care, where commonly used agents, such as opioids, NSAIDs, antidepressants, and anticonvulsants, exhibit wide variability in both effectiveness and toxicity (13). Among older adults with multimorbidity and/or polypharmacy, phenoconversion can either suppress metabolic activity, shifting a normal metabolizer toward a poor metabolizer phenotype, or accelerate metabolism, such as when inhibitors are discontinued or inducers are introduced (13, 31). Thus, phenoconversion is a central, yet likely underrecognized, driver of metabolic variability and may be a meaningful contributor to pain disparities (5, 6, 10–14, 28–31).

Opioids, such as codeine, tramadol, hydrocodone, and oxycodone differ in their dependence on CYP2D6-to generate their respective metabolites that contribute to analgesia. Codeine and tramadol are true prodrugs, requiring CYP2D6 enzyme activity to form morphine and o-desmethyltramadol, respectively. In contrast, hydrocodone is partially dependent on CYP2D6 to modulate pain through conversion to hydromorphone, while oxycodone's analgesic effect is enhanced by CYP2D6-mediated formation of oxymorphone. Consequently, unrecognized CYP2D6 phenoconversion can lead to inadequate pain control or, conversely, life-threatening toxicity manifested as respiratory depression.

Phenoconversion may also contribute to delirium risk in older adults (32). Opioid toxicity, analgesic failure or abrupt metabolic shifts can manifest as agitation or behavioral changes that may frequently be misinterpreted in individuals with multimorbidity or cognitive impairment. Though requiring further study, incorporating metabolic awareness into delirium evaluation may prevent misdiagnosis and inappropriate treatment escalation.

The following case-based illustrations are composites of clinical observations across multiple patients. Their purpose is to demonstrate the biological manifestation of phenoconversion and the clinical relevance of precision prescribing. Thus, the cases presented should not be interpreted as empirical validation that phenoconversion is a population-level driver of inequity.

### 3.1 Case 1: analgesic failure in a rural older adult

A 68-year-old male farmer with hypertension, hyperlipidemia, type 2 diabetes, chronic kidney disease with a baseline creatinine of 1.4 mg/dL, as well as depression treated with fluoxetine 40 mg daily, sustained an intertrochanteric hip fracture after falling from his tractor. Following cephalomedullary nail fixation surgical repair, he experienced severe postoperative pain that prevented mobilization. Despite the team's escalation of his hydrocodone to 10 mg every four hours, as well as subsequently transitioning him to oxycodone (similarly dosed: 10 mg six times daily), his pain remained poorly controlled, necessitating his transfer to an acute inpatient rehabilitation facility.

On admission to the acute inpatient rehabilitation facility, his medication list was reviewed and included amlodipine, lisinopril, metformin, empagliflozin, atorvastatin, and fluoxetine. Recognizing that fluoxetine is a strong CYP2D6 inhibitor (13, 31), which impacts activation of enzyme-dependent opioids through phenoconversion, the admitting physical medicine and rehabilitation physician discontinued CYP2D6-dependent opioids and initiated morphine sulphate, immediate release (7.5 mg for moderate pain and 15 mg for severe pain). The patient opted for the lower dose, since he was concerned about trying the 15 mg dose given morphine's historical reputation. Notably, the patient reported sleeping through the night after a single 7.5 mg dose and within 24 h achieved sustained analgesia on a total of 22.5 mg/day of morphine, a marked reduction when compared with the ineffective 60 mg/day of oxycodone (∼90 morphine milligram equivalents). With adequate pain control, he progressed rapidly in therapy and was discharged home within seven days, ambulating with a walker.

This case illustrates how unrecognized phenoconversion can prolong suffering, delay rehabilitation, and increase healthcare utilization. It also spotlights how a targeted adjustment can rapidly restore treatment effectiveness. Additionally, it features person-centered care and its core element, shared decision making (SDM), since the patient's concerns about morphine shaped the dosing strategy and contributed to treatment acceptance (33). Consistent with the Agency for Healthcare Research and Quality (AHRQ) SDM framework (33), aligning analgesic choices with patient values, functional goals and communication preferences is essential when managing pain in older adults with multimorbidity.

### 3.2 Case 2: respiratory arrest after CYP2D6 inhibitor discontinuation

A 72-year-old woman with hypertension, diabetes, chronic pain after multiple spine surgeries, and long-term oxycodone therapy (10 mg up to six times daily) was admitted to an acute care hospital with pneumonia and agitation. She was also taking bupropion, which is a strong CYP2D6 inhibitor, for chronic depression (13, 31). Concerned that bupropion might be contributing to her confusion, the inpatient team discontinued it. Her oxycodone dose was maintained at the family's insistence. Three days later, she was stable enough to transfer to an acute inpatient rehabilitation facility.

Three days after transfer, the patient experienced sudden respiratory collapse, which required three doses of naloxone for resuscitation. Review of her medication history revealed that the abrupt discontinuation of bupropion six days earlier, combined with its short half-life, had effectively reversed her CYP2D6 inhibition over the interval (13, 31). Her restored metabolic activity increased conversion of the parent drug, oxycodone, to its active metabolite, oxymorphone, exerting increased opioid effect that culminated in toxicity.

This case illustrates that removal of metabolic inhibition can unmask opioid potency, leading to life-threatening respiratory depression (13). Underscoring the critical safety implication, had the patient been taking fluoxetine (known for its long half-life) instead of bupropion, the washout interval that culminated in reversal of her poor metabolism could have placed phenoconversion after discharge, where her high risk for fatal apnea would have occurred at home without clinical monitoring. Thus, transitions of care should anticipate the potential for delayed metabolic shifts related to medication changes.

The family's involvement in analgesic decisions also highlights the importance of SDM and person-centered care (33). AHRQ's SDM framework supports structured conversations that help clinicians explain complex medication risks, including drug-drug-gene interactions, in ways that can promote patient and caregiver understanding and engagement (33). In turn, this facilitates input to align treatment choices with their values, preferences, and functional goals (33). These are critical considerations when selecting analgesics for older adults with multimorbidity, especially when metabolic shifts may alter opioid potency or safety. Incorporating SDM into polypharmacy precision prescribing workflows could help clinicians anticipate concerns and communicate risks related to phenoconversion, as well as ensure that analgesic decisions reflect what matters most to the patient.

Both cases highlight that phenoconversion-related risks may emerge during transitions of care, where medication changes, discontinuations, or formulary substitutions frequently occur. Rural older adults often transition between acute care, inpatient rehabilitation, and long-term care facilities, and each setting has varying levels of geriatric expertise. Thus, strengthening two-way communication, interdisciplinary training, and medication reconciliation across settings appears to be essential to prevent unrecognized metabolic shifts from contributing to analgesic failure or toxicity.

### 3.3 Transforming disparities into equities to impact pain outcomes for rural older adults

These cases demonstrate the bidirectional nature of phenoconversion (Figure 1). Metabolic inhibition can result in analgesic failure, while reversal of inhibition can precipitate toxicity (10–14, 34, 35). A single medication change, often routine in primary care, can produce profound shifts in clinical outcomes.

**Figure 1:**
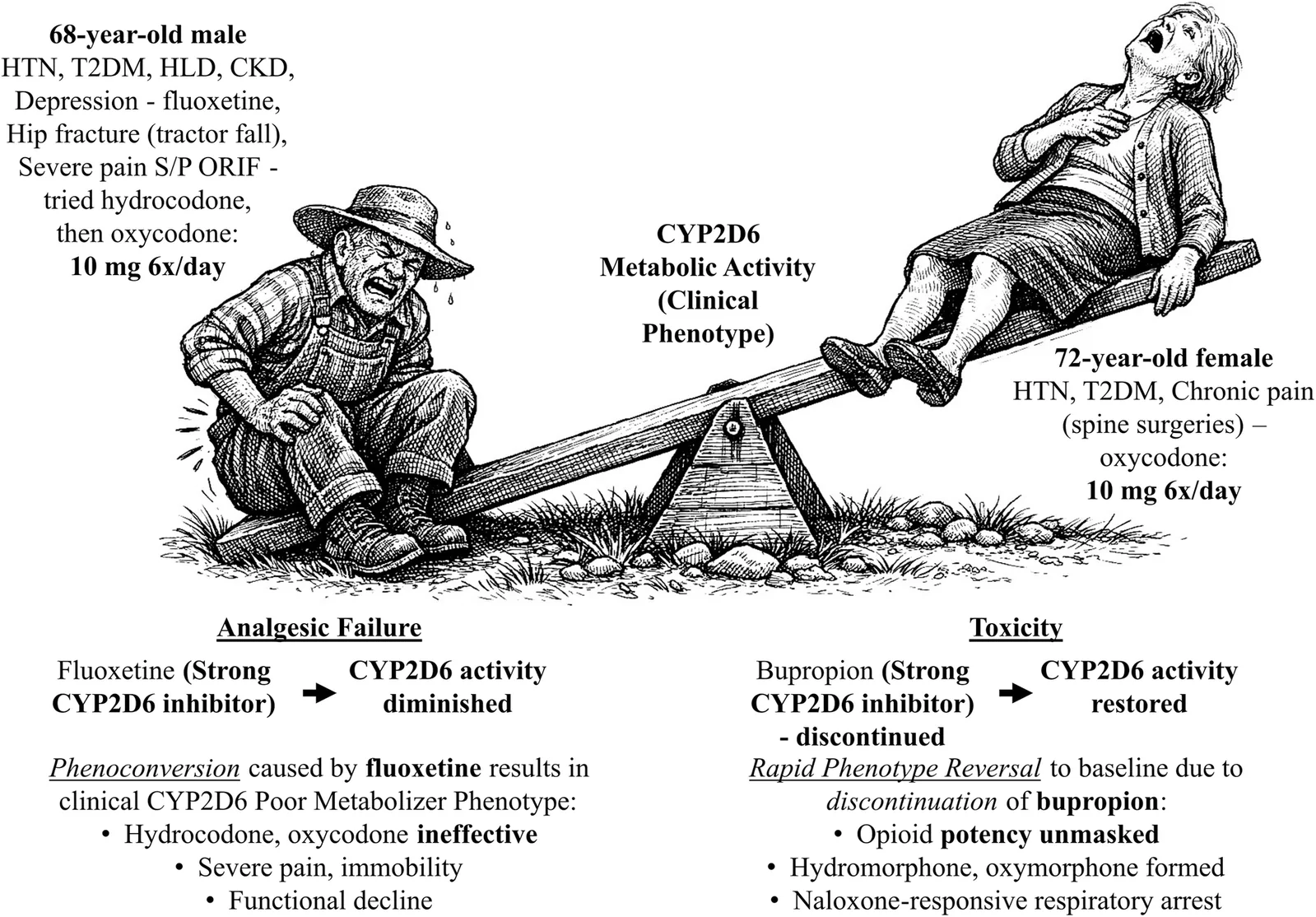
Bidirectional impact of phenoconversion in opioid metabolism. CYP2D6 inhibition reduces the bioactivation of enzyme-dependent opioids, contributing to inadequate analgesia (first case). Conversely, when inhibition is removed, metabolic activity rebounds, increasing opioid conversion to active metabolites and heightening toxicity risk, including respiratory depression (second case). HTN - hypertension, T2DM – type II diabetes mellitus, HLD - hyperlipidemia, CKD – chronic kidney disease, S/P ORIF - status-post open reduction and internal fixation surgery, CYP2D6 – cytochrome P-450 2D6 enzyme.

Older adults in rural environments have a lower age adjusted mortality than urban counterparts (22) and experience a disproportionate burden of pain within resource-limited clinical environments (1–8). They are among residents with a higher incidence of opioid prescriptions (20, 36) and among those with a higher incidence of medications that have PGx guidance (21). Older adults are especially vulnerable to metabolic fluctuations due to multimorbidity, polypharmacy, a higher incidence of PIMS, and age-related physiological changes (7), which structural inequities may amplify.

In rural settings, such vulnerability is likely compounded by limited access to specialists, pharmacists, and clinical decision support (CDS) tools, constraining clinicians’ ability to identify and manage metabolic variability in real time (4). When metabolic factors go unrecognized, clinicians may escalate ineffective therapies or inadvertently precipitate harm (4, 20). As a result, rural older adults may receive analgesic regimens that are mismatched with their functional metabolic profile, contributing to persistent pain, functional decline, and preventable adverse effects. These intersecting factors create conditions in which phenoconversion, a prevalent and clinically consequential mechanism (10–13), is a plausible contributor to medication-related harm that could interact with rural structural disadvantages, such as limited specialty access, workforce shortages, reimbursement constraints, and digital barriers. However, current evidence does not yet establish phenoconversion as a population-level mechanism producing inequity, underscoring the need for further research.

Fourth-generation pain disparities research calls for implementing and evaluating structural solutions that transform systems currently shaping pain outcomes for older adults. In rural settings, this transformation requires confronting overlooked root causes of persistent pain inequities. If these factors, such as the lack the infrastructure, staffing, and reimbursement support, exacerbate unrecognized metabolic variability and undermine guideline-concordant pain management, then treatment effectiveness and patient safety may be improved by embedding PGx-informed CDS (37), strengthening interdisciplinary collaboration, and aligning reimbursement with precision health services. These improvements could then be regarded as essential steps or pillars toward reducing inequities and improving pain outcomes for rural older adults. Building on that paradigm, the following implementation vignette offers an exemplar of how RHCs can serve as catalysts for innovation to advance equity in geriatric pain management.

## 4 A rural health clinic catalyzing innovation, an implementation vignette

Archer Family Health Care (AFHC), an RHC in Archer, Florida, reflects the demographic and clinical pressures facing RHCs caring for aging populations with complex needs. Florida has a disproportionate percentage of adults over age 65 (22%) compared to the national average (18%), and one in every 4.3 adults in Archer (171/740) is over age 65 (38, 39). High rates of PGx-relevant prescribing further underscore the importance of an equitable, precision-informed approach (21). Based on evidence indicating potential for positive outcomes (40–46), AFHC piloted a pragmatic model for integrating interdisciplinary precision prescribing services into routine care. Although insurance coverage for PGx testing varies by carrier, recent Florida legislation supported Medicaid coverage for biomarker testing (47), creating a timely opportunity to operationalize this precision health application.

The initiative addressed key implementation barriers, including limited direct pharmacist reimbursement that further restricted access to pharmacist services, uncertainty among clinicians about their own training needs, and clinicians' concerns about workflow disruption, by leveraging the existing Epic® electronic health record (EHR) system, which links AFHC to the broader UF Health network, along with the Epic® Genomics Module CDS (37). This infrastructure and a collaborative approach enabled pharmacists to use integrated medication records to generate personalized recommendations in a patient-friendly, educational format. Medication records included detailed clinician notes, intricate medication history, and PGx test results among pathology labs. When available, PGx results are linked to best-practice alerts that provide real-time reminders to clinicians. These resources equipped site clinicians to incorporate PGx-informed discussions into routine visits for patients whose insurance covered PGx testing, without increasing patient out-of-pocket costs.

Composite, early clinician impressions indicated that older adults appeared to value individualized explanations of their medications and seemed to trust precision-informed clinical decision making. Provider consensus appeared to reflect increased confidence in applying precision health principles within a busy primary care setting and overall satisfaction with the initiative. These perceived benefits do not represent formally evaluated research outcomes, as the initiative was designed as a quality improvement project. Therefore, perceived benefits should be interpreted as early experiential indicators rather than demonstrated effects.

This vignette illustrates how policy literacy, supportive reimbursement, interdisciplinary workforce engagement, and aligned workflows, can synergize to advance precision health in rural primary care. AFHC benefited from an earlier expansion of EHR infrastructure that supported needed access to pharmacists and PGx resources, which is an advantage not universally available in rural communities. Many RHCs lack CDS capable of identifying drug-drug and/or drug-gene interactions, leaving clinicians to manage complex polypharmacy without essential tools. Sustained and equitable adoption of precision-informed pain care and precision prescribing will require continued investment in infrastructure, workforce capacity, and supportive policy (4, 14, 15, 20). When these elements converge, RHCs can serve as catalysts for innovation, advancing a broader roadmap that is designed to empower patients, address intra-group variability, and strengthen equity in geriatric care.

## 5 A precision-informed roadmap to advance equity for rural older adults

Advancing equitable, safe, and effective care for older adults, starting with precision-informed pain care, requires a forward-looking strategy for those in rural communities. PGx, phenoconversion, and context-aware prescribing were integrated into routine, rural primary care in the implementation vignette. These elements provide an illustrative example of a visionary strategy where four pillars (Table 1) are implemented in combination: 1) embedding CDS tools; 2) leveraging telehealth to extend specialty expertise; 3) strengthening workforce competency in precision prescribing; and 4) alignment with a system-level policy to increase the potential for a sustainable precision health service in an RHC.

**Table 1:** Precision informed roadmap for advancing equity in rural geriatric pain management. This table summarizes the four pillars required to operationalize precision informed pain care in rural settings: 1) clinical decision infrastructure; 2) telehealth-enabled specialty access; 3) workforce capacity and training; and 4) system-level support (e.g., structural, digital, and policy). Pillars include core problems addressed, goals, actionable checklist items, and anticipated equity impacts, creating a system-level vision for integrating pharmacogenomics, phenoconversion awareness, and context aware prescribing into routine rural primary care.

Four pillars:	Clinical Decision Support (CDS), infrastructure	Telehealth and specialty access	Workforce capacity and training	System-level support - structural, digital and policy
Core Problems Addressed	Polypharmacy, multimorbidity, phenoconversion, unpredictable analgesic response.	Limited access to pain, pharmacy, geriatrics expertise in rural settings.	Workforce shortages and limited training in complex pharmacotherapy.	Reimbursement barriers, digital inequities, limited infrastructure.
Goals	Enable safer, individualized prescribing that accounts for metabolic variability.	Expand access to precision informed pain expertise despite geographic isolation.	Build clinician competency in PGx, phenoconversion, and complex pharmacotherapy.	Align reimbursement, digital infrastructure, and policy with precision health adoption.
Checklist	Routine medication reconciliation; EHR-integrated PGx results; CDS alerts for drug-drug interactions; CDS progression to drug-gene then to drug-drug-gene; Context aware dosing recommendations; Interoperability via HIE utilizing FHIR as an HL7 standard.	Tele-pain and tele-pharmacy consultation pathways; Virtual case reviews for complex regimens; Remote PGx interpretation support; CDS embedded in telehealth workflows	Case based learning on phenoconversion; SDM tools and interdisciplinary care models linking primary care, pharmacy, behavioral health; Precision prescribing training modules across primary, acute, rehab, and long-term care; Fellowship-supported implementation expertise	Broadband expansion and digital literacy support; Coverage for PGx testing and pharmacist services; Support for CDS and EHR integration; Value based metrics (falls, opioid AEs, functional recovery, readmissions); State level policy levers (e.g., Medicaid biomarker coverage); Regional HIE
Vision of Equity Impact	Real-time, context aware prescribing reduces avoidable adverse events and improves analgesic effectiveness for older adults with complex regimens.	Timely escalation and multimodal pain management despite geographic isolation.	Strengthens clinician readiness to deliver individualized, guideline-concordant pain care across settings.	Ensure sustainable, equitable adoption of precision-informed care in resource constrained rural clinics.

A consideration is that sites are not homogenous, and each must empirically strategize how to meet their specific needs by monitoring metrics that could include functional recovery, adverse events, time to treatment modification, among other measures (48). Operationalizing the proposed roadmap requires systems to define workflows for CDS alert review, involves pharmacists and telehealth consultation pathways, necessitates clinician training, financing mechanisms for PGx testing, and evaluation metrics such as the ability to modify time to treatment, rates for adverse events, and functional recovery. Because many rural clinics lack integrated EHRs, genomic modules, reliable internet, or embedded pharmacists, the roadmap includes low-technology alternatives, such as simplified medication review tools (e.g., AHRQ's Electronic Care Plan (49); and PharmDOG (50)), remote PGx interpretation, and tele-pharmacy consultation models.

### 5.1 Clinical decision infrastructure

**Problem:** Polypharmacy, multimorbidity, and phenoconversion create unpredictable analgesic response and toxicity risk for rural older adults, who have a documented higher rate of polypharmacy and PIMS than younger adults (7).

**Goal:** Since the equity outcome of interest is clinical benefit, individualized prescribing that accounts for metabolic variability, integrates SDM tools (33) and interoperable electronic care plans (eCare plans (49)), can strengthen clinical decision infrastructure for safer, optimized outcomes. The eCare plans allow patient goals, functional priorities, caregiver inputs, and medication histories (49) to accompany individuals across transitions of care settings where phenoconversion-related risks often emerge. Embedding SDM prompts and eCare plan elements into CDS workflows would ensure that context aware prescribing incorporates PGx data, medication data, and the patient's lived experience and treatment preferences.

Interoperability is essential for transmitting PGx results, medication histories, and CDS alerts across care settings. Rural clinics frequently lack interoperable EHR systems, yet electronic workflows are especially important in these settings where fragmented records and limited specialist access heighten risks related to unrecognized phenoconversion. Adoption of core standards for Health Information Exchange (HIE), such as the Fast Healthcare Interoperability Resources (FHIR), which is a Health Level Seven International (HL7) standard (51), would help ensure that drug-drug-gene interaction alerts follow patients across transitions of care to reduce the likelihood of missed metabolic shifts. When integrated EHR is not available, use of low-technology alternatives such as mobile phone app versions of eCare plans (49) and PharmDOG (50) should be explored.

**Checklist to get there:**

- Initiate routine medication reconciliation for patients, since both CDS and context-aware prescribing require accurate medication lists to function properly, introducing SDM and eCare plan concepts.
- EHR-integrated PGx results
- CDS alerts for drug-drug-gene interactions/phenoconversion risk
  1. When utilizing EHR-integrated CDS, begin with drug-drug alerts, which are commonly used.
  2. Progress to drug-gene alerts, which are increasingly utilized at institutions where PGx testing is routinely available to inform patient care.
  3. Next step: drug-drug-gene CDS, prioritizing opiates.
- Integration of context-aware dosing recommendations, ensuring interoperability.

**Vision of equity impact:** Real-time, context-aware prescribing that accounts for polypharmacy, multimorbidity, and phenoconversion, thereby reducing avoidable adverse events and improving analgesic effectiveness for older adults with complex regimens.

### 5.2 Specialty access & telehealth-enabled support

**Problem:** Rural clinicians lack access to pain, pharmacy, and geriatrics expertise.

**Goal:** Expand access to precision-informed pain expertise in resource-constrained rural settings to obtain clinical benefit. Intrinsically tied to the vision for telehealth and its implementation strategies in rural settings (52–54), this pillar is to be seen as a call to action to prevent widening of disparities that could be evident upon analysis of time to treatment modification, patient costs, pain control, functional recovery, adverse events, differences with rurality, socioeconomic position, insurance coverage, and digital access.

**Checklist to get there:**

- Tele-pain, tele-pharmacy consultation pathways
- Virtual case reviews for complex regimens
- Remote PGx interpretation support
- CDS embedded in telehealth workflows

**Vision of equity impact:** Timely escalation and multimodal pain management, despite geographic isolation, that overcomes rural clinicians’ lack of access to pain, pharmacy, and geriatrics expertise.

### 5.3 Workforce capacity and precision prescribing training

**Problem:** Workforce shortages, limited training in complex pharmacotherapy, fragmented records (e.g., when electronic records lack interoperability), lost patient treatment preferences and functional goals during transitions of care, all can precipitate undesired outcomes.

**Goal:** Create goal-aligned, optimized, and safer clinical benefits by building rural clinicians’ competency in PGx, phenoconversion, and complex pharmacotherapy to improve clinical benefits for older adults.

**Checklist to get there:**

- Utilize case-based learning related to phenoconversion and metabolic variability (55), where local cases are identified and developed as applied learning tools that are shared.
- Launch use of the SDM tools in the rural setting to help clinicians communicate risks and benefits of opioid options, incorporating patient and family preferences (33). Assess user uptake and ensure that needs for patients and caregivers are met during rollout, specifically to include assurance that communication needs are met for patients and caregivers with limited time availability, limited digital literacy, and limited digital access.
- Utilize interdisciplinary care models that link primary care, pharmacy, and behavioral health, sharing interoperable electronic systems whenever available.
- Create site-specific, precision prescribing training modules for the interdigitation of interdisciplinary teams across care settings, including primary care, acute care, inpatient rehabilitation, and long-term care, to ensure continuity and safe medication transitions, which incorporate SDM (33) and eCare plan tools across transitions (49).
- Rollout use of interoperable eCare plans (49) in the rural setting and assess user uptake as well as signs of improved coordination across primary care, acute care, rehabilitation, and long-term care settings, to ensure that patient goals and medication considerations are consistently communicated during transitions where phenoconversion risk may be heightened.
- Develop fellowship-supported implementation expertise to sustain and scale precision health practices (55–57)

**Vision of equity impact:** Strengthening clinicians’ readiness to deliver individually optimized, guideline-concordant care for the clinical benefit of older adults, including those with complex chronic conditions, in rural settings.

### 5.4 System-level support: structural, digital, and policy

**Problem:** Reimbursement barriers and digital inequities limit adoption of precision health practices that could realize clinical benefits for rural older adults.

**Goal:** Create the system-level conditions necessary for sustainable, precision-informed care that brings clinical benefits to rural older adults. As one example, reimbursement barriers intersect each pillar of the roadmap. Coverage for PGx testing pharmacist consultation, telehealth services, and CDS infrastructure impact the ability of rural clinics to implement precision-informed care. State-level policy changes, including Florida's Medicaid coverage of biomarker testing (47), illustrate how targeted reimbursement can expand access. Metrics such as reduced fall risk, fewer opioid-related adverse events, improved functional recovery, and lower readmission rates could serve as value-based levers for insurer adoption. Including reimbursement for use of SDM tools and interoperable eCare plans adds to equity-promotion, since these tools help ensure that precision-informed pain care brings benefit to all rural older adults, beyond those receiving care in better-resourced clinics.

**Checklist to get there:**

- Policies to develop digital health infrastructure to support hybrid care models
- Coverage for PGx testing as well as reimbursement-aligned workflows for pharmacist consultations and for precision health services, inclusive of clinician time developing interoperable eCare plans for patients (49) and utilizing SDM tools (33)
- Policies for CDS and EHR integration, such as measures to prevent alert fatigue and to track CDS guidance
- Regional HIE strategies that tailor implementation to site-specific RHC constraints, and quantify value-based metrics

**Vision of equity impact:** Creation of sustainable precision health delivery for the clinical benefit of older adults in resource-constrained settings and rural communities through effective address of structural barriers currently limiting adoption of precision health innovations.

## 6 Limitations

This perspective has several limitations. Direct evidence linking phenoconversion to rural population level inequities is limited. The illustrative cases cannot establish causality or generalizability. The implementation vignette was illustrative yet dependent on digital infrastructure to access specialized personnel. Since early experiential impressions were described, as opposed to formally evaluated outcomes in diverse settings, transferability to less-resourced clinics is currently uncertain. As the proposed roadmap depends on digital infrastructure, reimbursement, and specialized personnel that may be lacking in other rural settings, precision health interventions that are introduced unevenly could inadvertently widen inequities. Safeguards that include low technology alternatives, such as AHRQ's eCare plan (49) and PharmDOG (50), similarly must be evaluated by sites to determine their specific clinical benefit for rural older adults.

## 7 Discussion

Advancing clinical care equity in rural geriatric pain management requires addressing both the biological contributors to treatment variability and the structural conditions that shape clinical outcomes. Phenoconversion represents a clinically meaningful yet underrecognized mechanism through which multimorbidity, polypharmacy, and dynamic metabolic shifts alter analgesic effectiveness and toxicity. The cases presented illustrate how CYP2D6 inhibition or reversal can produce profound changes in opioid response, which may contribute to avoidable suffering, delayed recovery, and safety events. Although these cases cannot establish population-level causality, they highlight the clinical relevance of phenoconversion and underscore the need for system-level strategies that support context-aware prescribing in rural settings.

Phenoconversion also exposes broader limitations in current pain management models, such as limited clinician access to the tools, training, or real-time CDS needed to evaluate polypharmacy and metabolic risk. These gaps disproportionately affect older adults with multimorbidity, and complex medication regimens. Consequences of phenoconversion may be intensified in rural health systems facing persistent shortages of pain specialists, pharmacists, and geriatric expertise, along with limited access to advanced EHR infrastructure and CDS. Transitions of care, where medication changes and incomplete information exchange are common, create a source of added vulnerability.

To address the above gaps the proposed roadmap envisions four essential pillars: CDS, telehealth, workforce development, and system-level support, which were integrated within the RHC Implementation Vignette. Strengthening interoperability through participation in regional HIE, complete with standards such as the HL7 FHIR, can help ensure that pharmacogenomic results, medication histories, and drug-drug-gene alerts accompany patients across settings. Interoperable eCare plans that include SDM, and could include drug-drug-gene interactions, would further support continuity by preserving patient goals, caregiver perspectives, and functional priorities during transitions where metabolic shifts may emerge.

Telehealth-enabled pain, pharmacy, and PGx consultation pathways, can extend expertise to clinics lacking embedded specialists. However, digital equity strategies are needed to ensure these services do not widen disparities. Specifically, broadband expansion and support for patients with limited digital literacy are essential.

Workforce development remains a critical component of equitable implementation. Rural clinics require training not only in PGx and phenoconversion, but also in using CDS effectively. Additionally, training is needed to integrate SDM tools and eCare plans into routine practice and into interdisciplinary care coordination across primary care, acute care, rehabilitation, and long-term care settings. A fellowship that embeds precision prescribing, CDS alert interpretation, and risk communication, which is grounded in established competencies (55, 56), may accelerate implementation by building workforce capacity and system-level expertise.

Finally, policy alignment that includes reimbursement is essential for sustainable adoption. Coverage for PGx testing, pharmacist consultation, telehealth services, and CDS infrastructure determine whether rural clinics can implement precision-informed care. State-level policy changes, such as Medicaid coverage for biomarker testing (47), illustrate how targeted reimbursement can expand access. Value-based metrics, including reduced fall risk, fewer opioid-related adverse events, improved functional recovery, and lower readmission rates, could serve as levers for insurer adoption and policy advancement.

While evidence is needed to evaluate the impact of these approaches on disparity reduction at scale, the roadmap, grounded in biological plausibility and implementation experience, offers a strategic vision to advance safer, more effective, pain management for older adults. RHCs may even serve as a catalyst for this transformation when PGx-informed care is embedded within routine encounters and is supported by interdisciplinary collaboration and implementation-aligned processes. Rural systems might then begin to close long-standing equity gaps. Ultimately, improving geriatric pain outcomes is expected to require coordinated action across clinical, system and policy domains.

## Data Availability

The raw data supporting the conclusions of this article will be made available by the authors, without undue reservation.
